# Regulatory Role of miR-196a-5p in Angiogenesis-Related Markers in Endothelial Cells Exposed to Hypertensive Pregnancies

**DOI:** 10.3390/ijms27042047

**Published:** 2026-02-22

**Authors:** Aslah Nabilah Abdull Sukor, Nurul Iffah Mohd Isa, Nur Syakirah Othman, Azizah Ugusman, Mohd Faizal Ahmad, Nur Fariha Mohd Manzor, Shahidee Zainal Abidin, Amilia Aminuddin, Adila A. Hamid

**Affiliations:** 1 Faculty of Medicine, Universiti Teknologi Mara, Sungai Buloh Campus, Jalan Hospital, Sungai Buloh 47000, Selangor, Malaysia; aslahnabilah@uitm.edu.my; 2 Department of Physiology, Faculty of Medicine, Universiti Kebangsaan Malaysia, Cheras, Kuala Lumpur 56000, Malaysia; n.iffah233@gmail.com (N.I.M.I.); dr.nursyakirahothman@gmail.com (N.S.O.); dr.azizah@hctm.ukm.edu.my (A.U.); amilia@hctm.ukm.edu.my (A.A.); 3 Cardiovascular and Pulmonary (CardioResp) Research Group, Universiti Kebangsaan Malaysia, Bangi 43600, Selangor, Malaysia; 4 Department of Obstetrics and Gynaecology, Faculty of Medicine, Universiti Kebangsaan Malaysia, Cheras, Kuala Lumpur 56000, Malaysia; drmohdfaizal@ukm.edu.my; 5 Faculty of Medicine and Health Sciences, Universiti Sains Islam Malaysia, Nilai 71800, Negeri Sembilan, Malaysia; nurfariha@usim.edu.my; 6 Faculty of Science and Marine Environment, Universiti Malaysia Terengganu, Kuala Nerus 21030, Terengganu, Malaysia; shahidee.zainal@umt.edu.my

**Keywords:** hypertensive disorders of pregnancy, microRNA, HUVEC, endothelial dysfunction, angiogenesis

## Abstract

Offspring of hypertensive disorders of pregnancies (HDP) exhibit early-life endothelial dysfunction and have an elevated susceptibility to hypertension during adulthood which is potentially mediated by microRNA (miRNA), a key regulator of gene expression. RNA sequencing showed that miR-196a-5p was significantly upregulated in HUVEC exposed to HDP and may regulate angiogenesis in endothelial cells. Therefore, this study aims to elucidate the role of miR-196a-5p in regulating angiogenesis in HUVEC exposed to HDP. miR-196a-5p expression was validated by stem-loop RT-qPCR. Predicted target genes were identified using four algorithms, miRWalk, miRDB, TargetScan, and DIANA-microT-CDS, focusing on angiogenesis-related genes. Protein expression was confirmed through ELISA. Stem-loop RT-qPCR showed that miR-196a-5p expression was significantly upregulated in HDP HUVEC. Bioinformatic analysis revealed that the *PDGFRA* gene, a key regulator for angiogenesis, was significantly enriched. Overexpression of miR-196a-5p significantly downregulated *PDGFRA*, *VEGF*, and *bFGF* in HDP HUVEC, whereas its suppression upregulated these genes significantly. The ELISA result confirmed the corresponding changes at the protein level. However, *PDGFRA* protein levels increased with miR-196a-5p overexpression and decreased with its inhibition. Collectively, the results indicate that miR-196a-5p may have a regulatory effect on *PDGFRA*, *VEGF*, and *bFGF* that is associated with angiogenesis, and the modifications could be beneficial in future epigenetic targeted therapy.

## 1. Introduction

Hypertensive disorder of pregnancy (HDP) remains one of the leading causes of maternal and fetal morbidity and mortality globally, complicating approximately 5–10% of pregnancies [1]. Beyond its immediate impact on maternal and neonatal outcomes, HDP also has long-term consequences, as offspring born to affected mothers often experience alterations in endothelial function early in life, predisposing them to a higher likelihood of developing hypertension in adulthood [2].

HDP develops from the failure to achieve normal cardiovascular adaptations during pregnancy. These adaptations, including systemic vasodilation, increased plasma volume, and vascular remodeling, ensure adequate blood flow to the placenta. However, in HDP, improper cytotrophoblastic invasion disrupts these processes, resulting in abnormal remodeling of the uterine spiral arteries, which leads to placental hypoperfusion and ischemia [3]. Placental ischemia induces overproduction of the pro-angiogenic factor soluble fms-like tyrosine kinase-1 (sFlt-1), along with a decrease in anti-angiogenic factors such as placental growth factor (PlGF) and vascular endothelial growth factor (*VEGF*) [4]. As a soluble splice variant of VEGFR-1, sFlt-1 serves as a decoy receptor by sequestering *VEGF* and PlGF, thereby limiting their availability for angiogenesis [4]. This disruption in the balance between pro-angiogenic and anti-angiogenic factors impairs vascular remodeling, exacerbates placental dysfunction, and triggers systemic consequences. Placental dysfunction directly contributes to widespread endothelial dysfunction by inducing oxidative stress, inflammation, microthrombi, increased vascular permeability and vasoconstriction [5]. Collectively, these processes result in the hallmark hypertension observed in HDP.

While the maternal complications of HDP are significant, its effects extend beyond pregnancy, profoundly influencing fetal development. The same pathological mechanisms, such as disrupted angiogenesis and endothelial dysfunction, also impair vascular development in the fetus, increasing the risk of hypertension and long-term cardiovascular complications [6,7]. Human umbilical vein endothelial cells (HUVEC) serve as a valuable model for studying endothelial dysfunction, as they closely reflect the vascular environment in pregnancy. HUVEC derived from hypertensive pregnancies provide unique opportunity to investigate the cellular and molecular changes associated with HDP, including disruptions in angiogenesis and vascular remodeling. At birth, offspring of mothers with HDP exhibit impaired angiogenic capacity, evidenced by changes in circulating endothelial cells and umbilical vein-derived endothelial cells [8]. These disruptions are reflected in impaired microvascular development in infants exposed to HDP, with supporting evidence linking these changes to altered endothelial cell function [9]. These infants are more likely to experience microvascular dermal rarefaction, a reduction in the density of small blood vessels in the skin, during the first three postnatal months as their circulatory systems undergo remodeling [9]. This rarefaction has been associated with elevated blood pressure in young adults born to mothers with pregnancy complications [10]. Such findings suggest a mechanistic pathway connecting early-life endothelial dysfunction and abnormal vascular development with an increased risk of hypertension and cardiovascular disease later in life.

A growing body of evidence suggests that miRNAs play a critical role in mediating these vascular changes. By regulating key processes such as angiogenesis and vasculogenesis, miRNAs influence endothelial cell behavior and may contribute to the pathophysiological mechanisms of HDP. For instance, miR-335 and miR-195, which are significantly elevated in preeclamptic placentas, directly target key angiogenic factors such as PlGF and VEGF-A, respectively, disrupting pathways essential for proper vascular development [11]. Our previous miRNA profiling on HUVEC exposed to HDP revealed that miR-196a-5p was significantly upregulated followed by miR-199b-5p, miR-675-3p, miR-708-5p, miR-34c-5p, miR-153-3p, miR-145-3p, and miR-145-5p [12]. These findings also highlighted that these miRNAs, particularly miR-196a-5p, may play a key role in regulating angiogenesis in endothelial cells exposed to HDP. Therefore, further validation may reveal that the dysregulation of angiogenesis by miR-196a-5p contributes to the pathogenesis of HDP and its vascular complications. Moreover, findings from this study may provide insights into the molecular mechanisms underlying HDP and the potential role of selected miRNA as therapeutic targets for mitigating endothelial dysfunction.

## 2. Results

### 2.1. Clinical Characteristics of the Subjects

The study recruited subjects comprising eight normal pregnant women and eight patients diagnosed with HDP. The clinical characteristics of the mothers and their offspring are summarized in Table 1.

For the mothers, the median age in the normal group was 34 years (range: 29–39), while the HDP group had a median age of 35 years (range: 24–38). The racial distribution differed between groups, with the majority being Malay in the normal group (87.6%), while the HDP group had a more balanced racial representation (Malay 50%, Chinese 37.5%, Indian 12.5%). Both groups had similar parity distributions: 25% of mothers were primigravida (G1P0), and 75% were multigravida (>G1P0). A family history of hypertension was slightly more prevalent in the normal group (50%) compared to the HDP group (37.5%). In the HDP group, the average gestational age at diagnosis of hypertensive disorders was 22 weeks. Cesarean delivery occurred in 37.5% of the HDP cases, while no cesarean deliveries were reported in the normal group. Symptoms of preeclampsia in the HDP group included high blood pressure and the presence of urinary albumin.

For the offspring, the average gestational age at delivery was higher in the normal group (38.29 ± 0.57 weeks) compared to the HDP group (34.67 ± 1.42 weeks). The median birth weight was 2.89kg in the normal group and 2.49 kg in the HDP group. Delivery complications were observed in the HDP group and included preterm birth, small-for-gestational-age (SGA) infants, and low birth weight.

### 2.2. Validation of von Willebrand Factor (vWF) Expression in HUVEC

**Figure 1:**
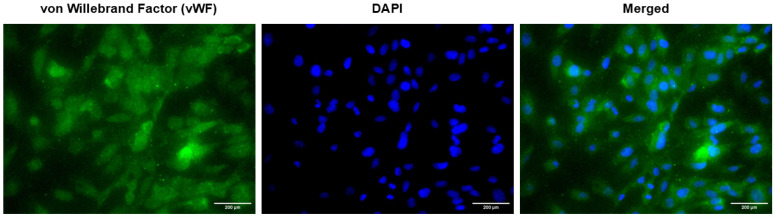
Expression of vWF in HUVEC isolated from the umbilical cord. Cells that were positively expressed vWF were stained with green color and the nucleus of the cells were stained with 4′,6-diamidino-2-phenylindole (DAPI) through ICC (×400 magnification). Scale bar of the pictures was set at 200 µm.

Since endothelial cells are known to express vWF, HUVEC isolated from the umbilical cord were stained with vWF using immunocytochemistry (ICC). Figure 1 shows that vWF was expressed in HUVEC positively under the fluorescence microscopy.

### 2.3. Target Genes Prediction of miR-196a-5p

**Figure 2:**
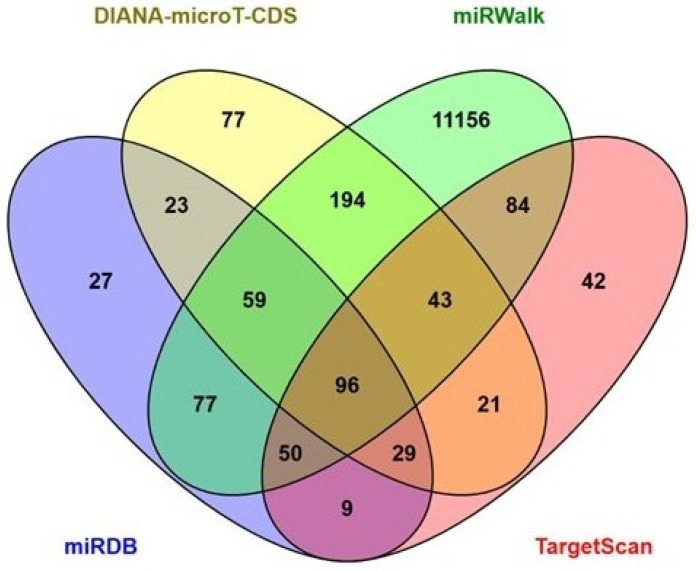
Venn diagram depicting the number of miR-196a-5p predicted target genes from miRDB, TargetScan, miRWALK, and DIANA-microT-CDS. Overlap indicates the number of genes predicted by more than one algorithm.

To understand the functional role of miR-196a-5p in hypertensive pregnancy, the prediction of target genes was identified using four data mining tools. Based on the analysis, a total of 11,987 genes were found to be targeted by miR-196a-5p. Of these, only 277 genes were predicted by at least three of the algorithms (Addition of 59 genes from miRDB, miRWalk and DIANA-microT-CDS, 43 genes from TargetScan, DIANA microT-CDS and miRWalk, 29 genes from TargetScan, miRDB and DIANA-microT-CDS, 50 genes from miRDB, TargetScan and miRWalk, 96 genes from all algorithms) (Figure 2). The list of genes for each database is listed in the Supplementary Table S1. To better visualize the relationships and functional clustering of these genes, a network representation was constructed using Cytoscape. This network illustrates the interactions among the genes and their associated functional categories, highlighting key hubs and interconnected pathways that may play a role in hypertensive pregnancy.

### 2.4. GO Analysis of Predicted Genes Targeted by miR-196a-5p

**Figure 3:**
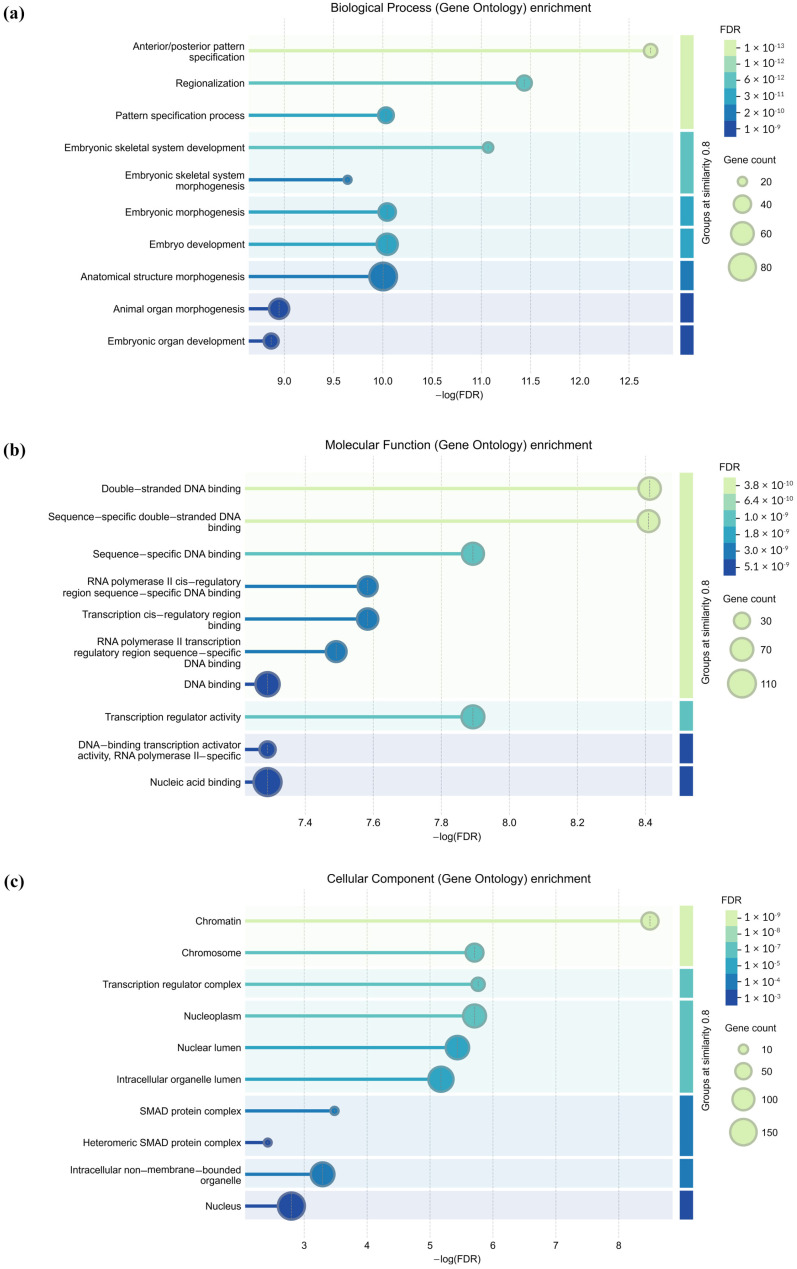
GO functional classification of miR-196a-5p. GO distributions of miR-196a-5p in the normal vs. HDP groups were classified into three categories: (**a**) cellular components, (**b**) molecular functions, and (**c**) biological processes.

To better understand the potential implications of miR-196a-5p, a total of 277 target genes were annotated using GO analysis, and their functions were categorized. The GO functional classification of miR-196a-5p is presented in the histogram and shown as the -log (false discovery rate) with the specification of the relevant biological process, molecular function and cellular component (Figure 3). Among the 10 biological processes involving the target genes of miR-196a-5p were anterior/posterior pattern specification, GO:0009952; regionalization, GO:0003002; pattern specification process, GO:0007389; embryonic skeletal system development, GO:0048706; embryonic skeletal system morphogenesis, GO:0048704; embryonic morphogenesis, GO:0048598; embryo development, GO:0009790; anatomical structure morphogenesis, GO: 0009653; animal organ morphogenesis, GO:0009887; and embryonic organ development, GO:0048568.

The molecular function category was enriched with 10 GO terms: double-stranded DNA binding, GO:0003690; sequence-specific double-stranded DNA binding, GO:1990837; sequence-specific DNA binding, GO:0043565; RNA polymerase II cis-regulatory region sequence-specific DNA binding, GO:0000 978; transcription cis-regulatory region binding, GO:0000976; RNA polymerase II transcription regulatory region sequence-specific DNA binding, GO:0000977; DNA binding, GO:0003677; transcription regulator activity, GO:0140110; DNA-binding transcription activator activity, RNA polymerase II-specific, GO:0001228; and nucleic acid binding, GO:0003676.

The cellular component category was enriched with five GO terms: chromatin, GO:0000785; chromosome, GO:0005694; transcription regulator complex, GO:0005667; nucleoplasm, GO:0005654; nuclear lumen, GO:0031981; intracellular organelle lumen, GO:0070013; SMAD protein complex, GO:007 1141; heteromeric SMAD protein complex, GO:0071144; intracellular non-membrane-bounded organelle, GO:0043232; and nucleus, GO:0005634.

Together, these findings suggest that miR-196a-5p may primarily influence gene expression programs by targeting transcriptional regulators and SMAD protein complexes involved in embryonic morphogenesis (GO:0048598), anatomical structure morphogenesis (GO:0009653), and embryonic organ development (GO:0048568). Through these developmental and morphogenetic processes, miR-196a-5p may indirectly regulate angiogenesis-related pathways, such as PI3K–Akt, MAPK, Wnt, and JAK–STAT signaling, which are critical for endothelial function in HDP.

### 2.5. KEGG Pathway Analysis of Predicted Genes Targeted by miR-196a-5p

**Figure 4:**
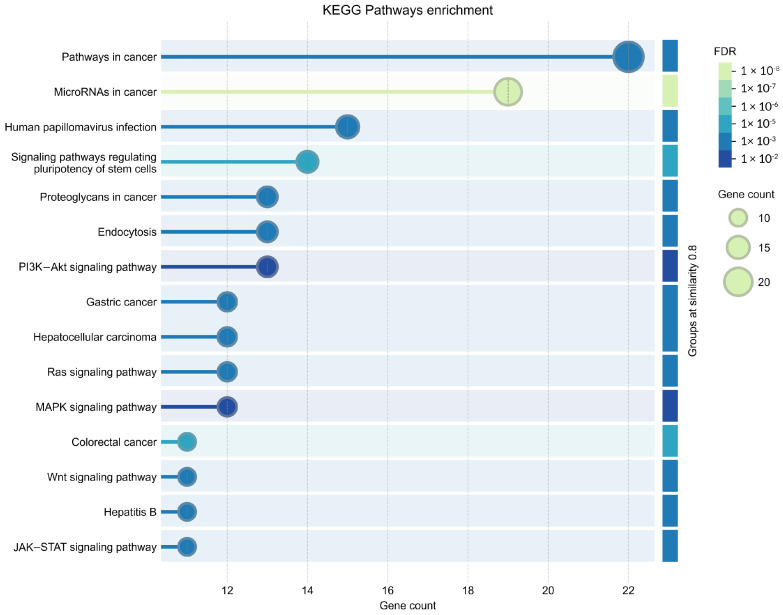
Bubble plot representing KEGG pathway enrichment analysis of miR-196a-5p target genes. The *x*-axis shows enriched KEGG pathways, and the *y*-axis indicates the gene count involved in each pathway. The size of the bubbles corresponds to the number of genes, while the color gradient represents the false discovery rate (FDR).

KEGG pathway enrichment analysis revealed that miR-196a-5p target genes were significantly associated with multiple signaling pathways, including pathways in cancer, PI3K–Akt signaling pathway, MAPK signaling pathway, Ras signaling pathway, Wnt signaling pathway, and JAK–STAT signaling pathway (Figure 4). Notably, several of these pathways are well-established regulators of angiogenesis, endothelial cell survival, migration, and vascular remodeling.

Among them, the PI3K–Akt signaling pathway plays a central role in angiogenic responses by regulating endothelial cell proliferation, nitric oxide production, and vascular permeability. Likewise, MAPK, Wnt, and JAK–STAT signaling pathways are critical mediators linking transcriptional regulation and developmental cues to angiogenic gene expression. Collectively, the GO and KEGG analyses suggest a coherent mechanistic framework in which miR-196a-5p targets transcriptional and developmental regulators that converge on angiogenesis-associated signaling pathways. Dysregulation of these interconnected networks may contribute to impaired angiogenesis and endothelial dysfunction observed in HDP.

### 2.6. Analysis of PPI Network and Hub Genes Regulated by miR-196a-5p

**Figure 5:**
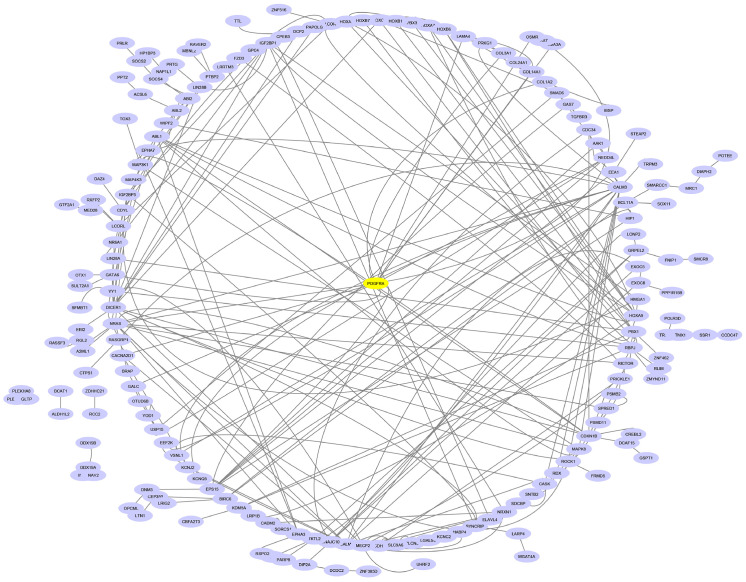
Cytoscape network visualization of the 277 target genes predicted for miR-196a-5p.

To elucidate the functional role of miR-196a-5p in HDP, a protein–protein interaction (PPI) network was constructed using Cytoscape to visualize the relationships among the 277 consistently predicted target genes (Figure 5). The network highlights the interconnected nature of these genes, showcasing their involvement in various biological and molecular processes.

**Figure 6:**
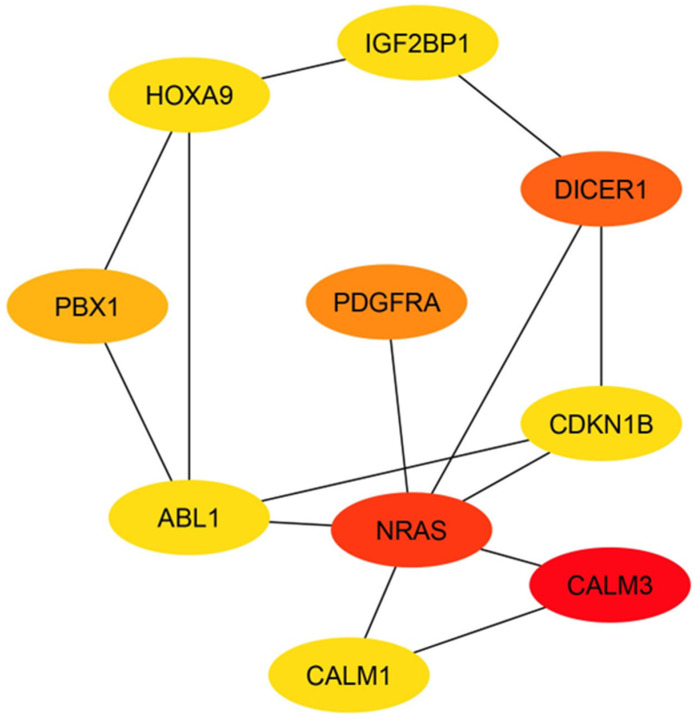
Top 10 hub genes in protein–protein interaction network of the predicted target genes of miR-196a-5p. Red to yellow CytoHubba nodes represent the highest- to lowest-ranked nodes based on the degree ranking method. *ABL1*, ABL proto-oncogene 1, non-receptor tyrosine kinase; *CALM1*, calmodulin 1; *CALM3*, calmodulin 3; *CDKN1B*, cyclin dependent kinase inhibitor 1B; *DICER1*, Dicer 1; *HOXA9*, homeobox A9; *IGF2BP1*, insulin like growth factor 2 mRNA binding protein 1; *NRAS*, neuroblastoma RAS viral oncogene Homolog; *PBX1*, PBX homeobox 1; *PDGFRA*, platelet derived growth factor receptor alpha.

The PPI network was further analyzed to identify hub genes, which are crucial nodes with high connectivity. Using CytoHubba’s degree ranking method, the top 10 hub genes with angiogenesis-related functions were identified (Figure 6). These hub genes represent key regulators within the network and include *NRAS*, *CALM3*, *PDGFRA*, *DICER1*, *CDKN1*, *CALM1*, *ABL1*, *PBX1*, *HOXA9* and *IGF2BP1*. Notably, the ranking of these nodes (from red to yellow) illustrates their relative significance, with *PDGFRA* emerging as the most central gene associated with angiogenesis.

**Figure 7:**
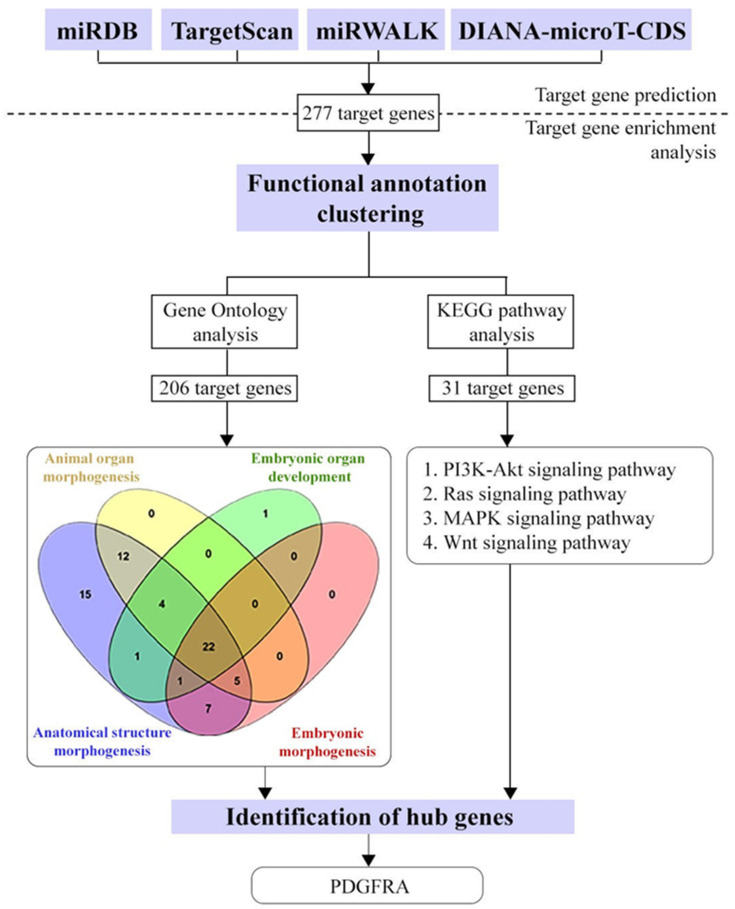
Integrated bioinformatic workflow for angiogenesis-related hub gene identification. Target genes were first predicted using four miRNA–target databases (miRDB, TargetScan, miRWalk, and DIANA-microT-CDS), yielding 277 common candidate genes. These genes were subjected to functional annotation clustering, including Gene Ontology (GO) and KEGG pathway enrichment analyses. Following enrichment, genes associated with angiogenesis-related biological processes and developmental pathways were retained for further analysis. GO analysis identified 206 genes involved in morphogenetic and developmental processes, while KEGG analysis highlighted 31 genes enriched in key signaling pathways relevant to angiogenesis, including PI3K–Akt, Ras, MAPK, and Wnt signaling. Integration of these results enabled the identification of *PDGFRA* as a central hub gene potentially mediating angiogenesis-related molecular mechanism.

To narrow the analysis to genes involved in angiogenesis, functional annotations were used to filter the network based on angiogenesis-related pathways, such as PI3K-Akt, MAPK, Wnt, and Ras signaling pathways. This selection highlighted genes directly linked to vascular development and remodeling processes crucial in HDP and this can be achieved through the prediction and functional annotation of miR-196a-5p (Figure 7). By focusing on angiogenesis-related genes, the analysis able to provide a deeper insight into the molecular mechanisms underlying hypertensive pregnancy and highlight the potential therapeutic targets within this critical biological process.

### 2.7. Expressions of miR-196a-5p, PDGFRA, VEGF, and bFGF in HUVEC Exposed to HDP

**Figure 8:**
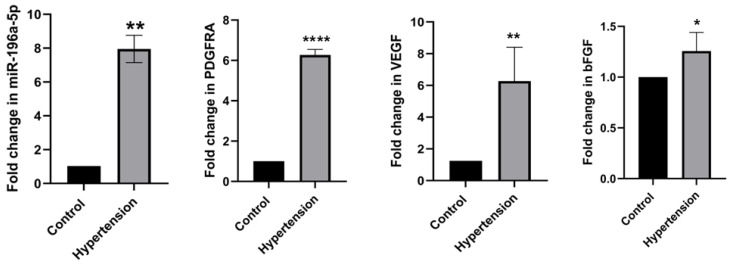
The expression levels of miR-196a-5p along with *PDGFRA*, *VEGF*, and *bFGF* in hypertensive HUVEC compared to normal HUVEC Values are presented as mean ± SEM. The asterisk denotes significant differences between hypertensive and normal HUVEC at * *p* < 0.05, ** *p* < 0.01 and **** *p* < 0.0001 with n = 6.

Based on previous RNA sequencing analysis, miR-196a-5p emerged as a strong candidate for its potential role in vascular biology and endothelial dysfunction, both of which are pivotal in the progression of HDP. To validate the expression of miR-196a-5p in HUVEC, stem-loop qPCR analysis confirmed that miR-196a-5p was significantly upregulated by 7.7-fold in HUVEC exposed to HDP compared to normal HUVEC (*p* < 0.01, *p*-value = 0.0011) (Figure 8). This validation strongly aligns with our previous RNA sequencing finding, reinforcing the robust expression changes in miR-196a-5p and its potential involvement in the pathophysiology of HDP.

Based on in silico analysis, we have selected three genes that are associated with angiogenesis. These genes, known as *PDGFRA*, *VEGF*, and *bFGF*, were found to be significantly upregulated by 6.27-fold (*p* < 0.0001), 6.27-fold (*p* < 0.001, *p*-value = 0.0013) and 1.26-fold (*p* < 0.05, *p*-value = 0.0163), respectively, in hypertensive HUVEC (Figure 8). These shown that upregulation of *PDGFRA*, *VEGF*, and *bFGF* might be significant in regulating angiogenesis in HUVEC exposed to HDP.

### 2.8. Regulation of miR-196a-5p in HUVEC Exposed to HDP and Its Predicted Target Genes

**Figure 9:**
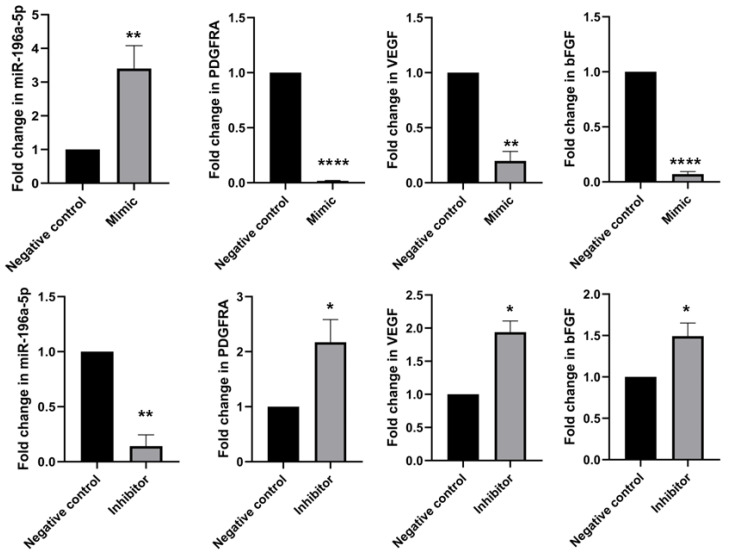
Expressions of miR-196a-5p and its target genes, *PDGFRA*, *VEGF*, and *bFGF* in HUVEC post-transfection with miR-196a-5p mimic and inhibitor against negative control through RT-qPCR. Values are shown as mean ± SEM, n = 6, with * *p* < 0.05, ** *p* < 0.01, and **** *p* < 0.0001.

**Figure 10:**
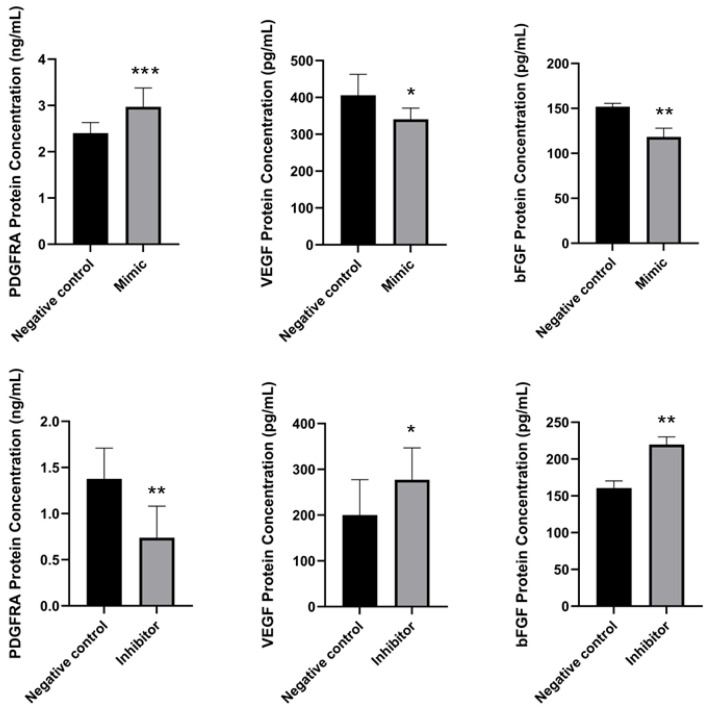
The figure shows the protein expression of *PDGFRA*, *VEGF*, and *bFGF* in hypertensive HUVEC significantly compared to control samples in the presence of miR-196a-5p mimics and inhibitors vs. negative controls respectively using the ELISA method. Values show mean ± SEM, n = 6 with * *p* < 0.05, ** *p* < 0.01, and *** *p* < 0.001.

To further determine the association between miR-196a-5p expression and *PDGFRA*, *VEGF*, and *bFGF* genes, HUVEC were transfected with miR-196a-5p mimic and inhibitor. After the transfection, the levels of *PDGFRA*, *VEGF*, and *bFGF* were measured using RT-qPCR. In mimic transfection, the expression of *PDGFRA*, *VEGF*, and *bFGF* were significantly downregulated as compared to the negative control (*p* < 0.0001, *p* < 0.01, *p* < 0.0001, respectively) (Figure 9). Conversely, the suppression of miR-196a-5p resulted in a substantial increase in the expression of *PDGFRA*, *VEGF*, and *bFGF* when compared to the negative control (*p* < 0.05). These results indicate that miR-196a-5p has a crucial role in regulating the expression of these genes in angiogenesis. Additionally, the ELISA analysis of protein expression demonstrated that the level of *VEGF*, and *bFGF* corresponded to the pattern of their mRNA, where the protein concentration of *VEGF*, and *bFGF* reduced significantly as miR-196a-5p mimic was transfected into HUVEC (*p* < 0.05 and *p* < 0.01, respectively) and increased significantly for miR-196a-5p inhibitor in both proteins (*p* < 0.05 and *p* < 0.01, respectively). However, the expression of *PDGFRA* showed the opposite trend, where the protein concentration level increased with the presence of miR-196a-5p mimic but decreased with miR-196a-5p inhibitor (*p* < 0.001 and *p* < 0.01, respectively) (Figure 10).

## 3. Discussion

miRNAs play a crucial role in maintaining physiological cell equilibrium by regulating gene expression. HDP have been linked to miRNA dysregulation. Altered miRNA expression may affect immune tolerance or angiogenesis, resulting in increased vascular resistance. Studies have found that preeclampsia is consistently associated with dysregulation of several miRNAs, such as miR-155 and miR-210 [13]. Hromadnikova et al. observed alterations in miRNAs in placental tissues associated with cardiovascular and cerebrovascular disease [14]. These results highlighted that miRNA dysregulation may be a crucial factor in the development of HDP and may have potential as biomarkers.

The clinical profile of the HDP cohort in the present study highlights the potential for significant miRNA-driven vascular changes, indicative of a high-severity, early-onset phenotype. Participants in the HDP group were diagnosed at a median of 22 weeks’ gestation, a period typically linked with significant placental insufficiency and modified molecular signaling. The clinical severity was evidenced by maternal systolic blood pressures ranging from 140 to 160 mmHg at diagnosis [15], while heart rate and blood pressure remained elevated through delivery, reflecting persistent hemodynamic stress [16]. The maternal factors were directly linked with adverse neonatal outcomes; although the median birth weight difference was 0.4 kg (2.49 kg in HDP vs. 2.89 kg in control group), the impact is significant when considering the high prevalence of preterm birth and Small for Gestational Age (SGA) infants. Furthermore, the 37.5% cesarean delivery rate aligns with global trends for medically indicated interventions in early-onset HDP [17]. The characteristics examined include diagnostic vitals to neonatal outcomes validate that the HUVEC samples analyzed represent a clinically robust model of HDP pathology.

In this study, vWF staining confirms that the cells maintained in culture are endothelial and exhibit an endothelial phenotype. However, this approach does not demonstrate the preservation of disease-specific endothelial characteristics associated with hypertensive disorders of pregnancy. Although endothelial identity appears to be retained, the extent to which pathological features related to hypertensive pregnancies persist over successive passages remains unclear. This should be noted as a limitation when interpreting the results. Further investigation of endothelial characteristics specific to hypertensive pregnancies across multiple passages would therefore be of interest and should be considered in future studies.

Based on previous sequencing findings, eight highly expressed miRNAs in hypertensive HUVEC were identified [12]. miR-196a-5p was selected as it was the most upregulated miRNA among others in hypertensive HUVEC. The finding of this miRNA on its role in regulating angiogenesis is correlated with the previous study that indicates overexpression of miR-196a inhibits *ANXA1*, which is an angiogenesis mediator, and dysregulates cell migration, formation and branching of capillary-like structures, and inhibits blood vessel formation in pregnancy related to angiogenesis in endothelial cells [18]. Tong et al. reported that elevated expression of miR-196a-5p also reduced vascular smooth muscle cell proliferation, oxidative stress and vascular remodeling in hypertensive rats via *BACH1* suppression [19]. These studies demonstrate that dysregulation of miR-196a-5p led to angiogenesis defects.

In this study, we demonstrated that the expression profile of miR-196a-5p in hypertensive HUVEC correlated with the targeted genes of angiogenesis. *VEGF*, *bFGF*, and *PDGFRA*, which are involved in the PI3K-AKT pathway were selected. The PI3K-AKT pathway is implicated in the negative regulation of angiogenesis in pregnancy hypertension [20,21]. *VEGF* and *bFGF* were identified as target genes using four in silico miRNA–target prediction databases (miRWalk, miRDB, TargetScan dan DIANA). *PDGFRA*, which regulates angiogenesis, was selected by using the Cytoscape platform. We observed significant upregulation of *PDGFRA*, *VEGF*, and *bFGF* in HUVEC exposed to HDP through RT-qPCR.

Genes involved in the PI3K pathway in angiogenesis include *PDGFRA*, *VEGF*, insulin-like growth factor-1 receptor (*IGF-1R*), *bFGF*, and angiopoietin [22,23]. In addition to AKT, identified as the main target of PI3K, some studies have found the involvement of other genes in this pathway, including the *PDGFRA*, *VEGF*, *IGF*-*1R*, *bFGF*, and angiopoietin in the formation of angiogenesis associated with the site PI3K pathway [23]. Gródecka-Szwajkiewicz et al. reported that a decrease in the main angiogenic proteins in preterm birth, namely *VEGF* and *FGF*, reflects abnormal placental vascular changes and is associated with cardiovascular disease [24]. *PDGFRA* induces angiogenesis by regulating *VEGF* production and modulating perivascular cell proliferation and recruitment [25].

It is imperative to conduct gain and loss of miRNA function tests to assess the effect of overexpression of miR-196a-5p [26]. Based on Figure 8, we discovered that miR-196a-5p, *bFGF*, *PDGFRA*, and *VEGF* are upregulated in HUVEC exposed to HDP. However, our transfection results substantiated that overexpression of miR-196a-5p downregulated *PDGFRA*, *VEGF*, and *bFGF*, and vice versa for the suppression of miR-196a-5p through the transfection of miR-196a-5p’s mimic and inhibitor, respectively. Although miR-196a-5p expression was elevated in HUVEC exposed to HDP, angiogenic genes such as *VEGF*, *PDGFRA*, and *bFGF* were also upregulated, indicating that strong disease-associated transcriptional activation can override or coexist with miRNA repression. miRNAs primarily function as post-transcriptional fine-tuners rather than dominant suppressors of gene expression, modulating translation and mRNA stability rather than completely silencing targets [27]. In pathological conditions such as HDP, powerful upstream signals (e.g., hypoxia, inflammation, oxidative stress) drive the transcription of pro-angiogenic factors, and miRNA increases may reflect a compensatory response that is insufficient to fully counteract transcriptional induction [28]. In addition, this in vitro investigation revealed that the downregulation of these targeted genes in the presence of miR-196a-5p overexpression occurred solely at the mRNA level. In line with previous studies, investigated the interaction between miR-196a and *VEGF* expression and angiogenesis characteristics in HUVEC [18]. This showed that overexpression of miR-196a in HUVEC could downregulate the expression of Annexin-A1 (*ANXA1*). *ANXA1* is a mediator of *VEGF* that reduces cell migration, and affects capillary formation and neovascularization. Although miR-196a-5p able to decrease the expression level of *VEGF* in HUVEC, there is a possibility that *VEGF* is not a direct target of miR-196a-5p. This may occur when a miRNA targets many transcripts of the target gene, and each transcript can be targeted by many miRNAs through the attachment of its seed site to the 3′-untranslated region (3′-UTR) of the gene [29,30]. Through this attachment, mRNA degradation or translation can be repressed. Therefore, miR-196a-5p may act as a regulator in hypertension during pregnancy.

However, differences in protein expression are observed when transfection is performed. The presence of miR-196a-5p suppresses *VEGF* and *bFGF* protein levels but increases *PDGFRA* protein levels. For *PDGFRA* protein, translational activation occurs via a non-canonical pathway, in which the nuclear state of miRNA induces epigenetic changes, indirectly activating gene transcription [31]. In the cytoplasm, the miRNA-binding protein AGO2 is required to produce high protein levels [32]. miRNAs can activate translation by binding to different target sites in various sub-cellular locations under different cellular conditions [33].

Several aspects of this study could be improved. First, biological validations such as transwell migration assay, tube formation assay, and wound healing assay may enhance the understanding of the miR-196a-5p on angiogenesis in endothelial cells exposed to HDP, particularly when the cells are transfected with miR-196a-5p mimic and inhibitor. Furthermore, the association between miR-196a-5p and *PDGRFA*, *VEGF*, and *bFGF* can be confirmed by conducting a luciferase reporter assay. This assay will help to validate the interaction of miR-196a-5p with its target genes. In addition, the level of protein expressions can be further validated through Western blot, as Western blot is a gold standard method to visualize and quantify the protein level in the samples. Additionally, although *VEGF* and *bFGF* protein levels were measured in cell lysates, both are secreted factors, and analyzing only the cell lysates may not fully represent endothelial activity. Measuring *VEGF* and *bFGF* levels in the culture supernatants would provide a more complete understanding of their effects on angiogenesis. In this study, endothelial angiogenic function was inferred primarily from in silico analysis and gene expression changes following miR-196a-5p modulation, which alone cannot fully define functional angiogenic capacity. The functional capacity of endothelial cells and capillary-like network formation were not directly assessed. Future studies should therefore include functional assays, such as Matrigel tube formation assays using HUVEC to further validate the effects of miR-196a-5p on endothelial function.

## 4. Materials and Methods

### 4.1. Subject Recruitment

This study involving human tissue collection was approved by the Universiti Kebangsaan Malaysia Research Ethics Committee (project code: JEP- 2020-195). All patients provided signed informed consent. Sixteen subjects were recruited from Hospital Canselor Tuanku Muhriz Universiti Kebangsaan Malaysia comprising eight patients with HDP and eight normotensive pregnant women as controls. The study included both primiparous and multiparous women. Inclusion criteria required participants to be at least 20 weeks into gestation and willing to participate in this study. For those classified un- der HDP, diagnostic criteria included a systolic blood pressure of ≥140 mmHg and/or a diastolic blood pressure of ≥90 mmHg, with/without proteinuria after 20 weeks of gestation, with resolution to baseline by 12 weeks postpartum. For normal pregnant women, the criteria were pregnancy with normal blood pressure (<140/90 mmHg), absence of proteinuria, and no medical or obstetric complications.

Exclusion criteria for all subjects included pregnant women unwilling to, pregnant women who were already hypertensive, with chronic hypertensive conditions, preeclampsia or eclampsia, gestational diabetes mellitus, autoimmune disorders such as systemic lupus erythematosus or rheumatoid arthritis, or infectious diseases such as human immunodeficiency virus or hepatitis B virus. In addition, any fetus with abnormal congenital or chromosomal diagnoses before or after delivery was excluded from the study.

### 4.2. HUVEC Isolation and Culture

HUVEC were isolated from human umbilical cords using an enzymatic technique as described in a previous study [34]. Umbilical cords were cleaned and treated with 0.01% (*w*/*v*) collagenase (Worthington Biochemical Corporation, Lakewood, New Jersey (NJ), United States of America (USA)) to isolate the HUVEC. The isolated cells were cultured in endothelial cell medium (ECM) supplemented with 5% fetal bovine serum, 1% endothelial cell growth supplement, and 1% penicillin-streptomycin (ScienCell Research Laboratories, Carlsbad, CA, USA) at 37 °C in a humidified incubator with 5% carbon dioxide (CO_2_). The culture medium was replaced every other day until the cells reached confluence. All experiments were conducted using HUVEC at passages 3–4 with 80% confluency.

### 4.3. Validation of vWF Expression in HUVEC Through Immunocytochemistry (ICC)

Determination of vWF expression in HUVEC isolated from the umbilical cords was performed using the ICC method according to Mohd Isa et al. [12]. A total of 3 × 10^4^ cells/well were cultured on 12 mm poly-L-lysine-coated coverslip (Electron Microscopy Sciences, Hatfield, PA, USA) inside of a 24-well plate (NEST, Wuxi, China) until the cells had reached 60% confluency. Cells were stained according to Anti-rabbit IgG-FITC Immuno Fluorescence Staining Kit instructions (Elabscience, Houston, TX, USA), and 0.025 mg/mL vWF polyclonal antibody (Elabscience, Houston, TX, USA) was used as the primary antibody. The vWF expression in HUVEC was observed using Carl-Zeiss™ Axio Vert. A1 inverted microscope (Carl-Zeiss, Oberkochen, Germany).

### 4.4. Total RNA Extraction

Total RNA was extracted from HUVEC using the miRNeasy kit (Qiagen, Germantown, MD, USA) following the protocol provided. The extracted total RNA was evaluated using the RNA Nano 6000 Assay Kit of the Agilent Bioanalyzer 2100 system (Agilent Technologies, Santa Clara, CA, USA) to determine its integrity, purity, and concentration.

### 4.5. Validation of miR-196a-5p with Stem-Loop Reverse Transcription Quantitative Polymerase Chain Reaction

To validate the RNA sequencing data, the expression of miR-196a-5p, identified as significantly differentially expressed and associated with angiogenesis in HDP, was analyzed using stem-loop Real-Time-quantitative Polymerase Chain Reaction (RT-qPCR). A total of 1 µg of small RNA-enriched total RNA was synthesized using an MMLV Reverse Transcriptase 1st-Strand cDNA Synthesis Kit (Biosearch Technologies, Hoddesdon, United Kingdom (UK)) with an additional 0.1 µM of the stem-loop primer. Prior to RT-qPCR, pre-PCR of miR-196a was performed in a 10 µL of total reaction consisting of 5 µL of 2× miRCURY SYBR Green Master Mix, 0.1 µM of each forward and reverse primer, and 3 µL of the synthesized cDNA (diluted 1:60). The pre-PCR and qPCR were carried out according to a previously published protocol [35]. All primer sequences to determine the expression of miR-196a-5p in the samples were followed according to Mohd Isa et al. [12].

All reactions were prepared in a 96-well plate format, and qPCR was performed using a CFX96™ Real-Time PCR detection system (Bio-Rad, Hercules, CA, USA). The reactions were conducted under following thermocycling conditions: initial denaturation at 95 °C for 10 min, followed by 45 cycles of amplification at 95 °C for 10 s, annealing at 59.2 °C for 30 s, and elongation at 72 °C for 10 s, with an additional elongation step at 40 °C for 1 s. The reaction kinetics of each primer set and protocol were verified with the melting profile. Amplification signals were acquired during the elongation step using Bio-Rad CFX Manager software version 3.1. The threshold cycle (CT) value was used to calculate miRNA expression. The U6 gene was used to normalize the quantitative analysis. Relative miRNA expression was calculated using the 2^−∆∆CT^ method, with ∆∆CT = CT of miR-196a-5p − CT of U6.

### 4.6. In Silico Analysis

#### 4.6.1. Prediction of miR-196a-5p Target Genes

To predict the target genes of miR-196a-5p, four bioinformatic tools were used: (1) miRWalk algorithm (http://mirwalk.umm.uni-heidelberg.de/, accessed on 18 December 2024), (2) miRDB v5.0 (http://mirdb.org, accessed on 18 December 2024), (3) DIANA-micro-T-CDS (https://dianalab.e-ce.uth.gr/html/dianauniverse/index.php?r=microT_CDS, accessed on 18 December 2024), and (4) TargetScan (http://www.targetscan.org, accessed on 18 December 2024). Predicted genes were selected according to the parameters set for each bioinformatics tool. In the miRWalk algorithm, genes with a score greater than 0.95 were used as input for the functional enrichment analysis [36]. For miRDB, predicted targeted genes were selected based on a predicted score ranging from 50 to 100 [37]. In the DIANA-micro-T-CDS algorithm, predicted target genes were selected using a threshold score of 0.6 [38]. In TargetScan, predicted genes were selected with a context ++ score less than −0.04 [39]. Overlapping predicted genes from at least three of these algorithms were identified and visualized using a Venn diagram. These overlapping genes were then subjected to downstream functional and pathway analyses.

#### 4.6.2. Gene Ontology and KEGG Pathway Enrichment Analyses

The genes targeted by at least three algorithms were analyzed using the STRING database (http://string-db.org, accessed on 18 December 2024) for functional annotation and clustering. Functional enrichment visualization was conducted under the biological process, molecular function and cellular component for Gene Ontology categories, grouping terms with a similarity threshold of ≥0.8. This analysis aimed to identify the biological roles and pathways associated with miR-196a-5p target genes, particularly those relevant to angiogenesis.

#### 4.6.3. Construction of the PPI Network and Identification of Hub Genes

The protein–protein interaction (PPI) network of the overlapping target genes was constructed using the STRING database. The network was then imported into Cytoscape software (version 3.10.3), and hub genes were identified using the CytoHubba plugin. The top 10 hub genes were selected based on the degree method, and their relevance to angiogenesis-related pathways was evaluated.

### 4.7. Transient miR–196a–5p Transfection

Transfection was performed using Lipofectamine RNAimax (Invitrogen, Carlsbad, CA, USA) with Opti-MEM I medium without serum or antibiotic. In this study, miR-196a-5p mimic and inhibitor were used to investigate the functional role of this miRNA. The HUVEC were transfected with 50nM miR-196a-5p mimic or negative control mimic (Qiagen, USA). For the miR-196a-5p mimic, HDP HUVEC were seeded in complete ECM in a 6-well plate at 2.85 × 10^5^ cells/well overnight until they reached 80% confluency. For the miR-196a-5p inhibitor, HDP HUVEC were transfected during the suspension stage at 6 × 10^5^ cells/well with 50 nM of miR-196a-5p inhibitor or negative control inhibitor. The sequences of the miR-196a-5p mimic and inhibitor used for the experiment are listed in Table 2. After 4 h of transfection, the medium was replaced with complete ECM and incubated for 24 h at 37 °C with 5% CO_2_. The efficiency of transfection was determined using a BD FACSAria II flow cytometer (BD Biosciences, San Jose, CA, USA) (Supplementary Figures S2 and S3).

### 4.8. Determination of Target Gene Expression in HUVEC Using RT–qPCR

The mRNA expression of *PDGFRA*, *VEGF* and *bFGF* was quantified using RT-qPCR, following a previously established method [40]. Complementary DNA (cDNA) was synthesized using an MMLV Reverse Transcriptase 1st-Strand cDNA Synthesis Kit (Biosearch Technologies, Hoddesdon, UK), according to the instructions provided. The qPCR primers were designed using Primer3 software (https://primer3.ut.ee/, accessed on 15 November 2020) based on sequences available in the NCBI GenBank database (Table 3). Glyceraldehyde-3-phosphate dehydrogenase (*GAPDH*) served as the internal control for normalization. The qPCR was performed on a Bio-Rad CFX96 cycler (Bio-Rad Laboratories, Hercules, CA, USA), using ViPrime-PLUS Taq qPCR Green Master Mix I (SYBR^®^ Green Dye) (Vivantis, Malaysia) with an initial denaturation at 95 °C for 2 min, followed by 40 cycles at 95 °C for 15s, 60 °C for 1 min and a final cooling step at 4 °C. The reaction kinetics of each primer set and protocol was verified with the melting profile. The relative expression of *PDGFRA*, *VEGF* and *bFGF* was determined using the 2^−∆∆CT^ method, where ∆∆CT = CT of target gene − CT of *GAPDH*.

### 4.9. Measurement of Target Protein Levels in HUVEC Using Enzyme-Linked Immunosorbent Assay (ELISA)

The level of *PDGFRA*, *VEGF*, and *bFGF* proteins were quantified using Human *PDGFRA*, *VEGF* and *bFGF*/FGF2 kits (Elabscience, Houston, TX, USA), following the manufacturer’s protocol. HUVEC lysates were used for protein measurement. The ELISA involved adding samples and standards in duplicate to a 96-well antibody-coated plate, followed by incubation at 37 °C for 90 min. After removing the contents, biotin-labeled detection antibody was added and incubated for 1 h at 37 °C, followed by three washes. HRP-conjugate was then applied in the dark, incubated at 37 °C for 30 min, and washed thoroughly five times. Subsequently, a substrate reagent was added, incubated in the dark at 37 °C for 15 min, and the reaction was stopped with a stop solution. Absorbance was measured at 450 nm using a microplate reader, and a standard curve was generated from the absorbance of the standards to facilitate data analysis.

### 4.10. Statistical Analysis

Statistical analyses were performed using GraphPad Prism 5.0 (GraphPad Software, Inc., La Jolla, CA, USA). Statistical significance between groups was assessed using Student’s *t*-test. A *p*-value of less than 0.05 was considered statistically significance. All data are expressed as mean ± standard error of the mean (SEM).

## 5. Conclusions

In summary, our findings demonstrate that miR-196a-5p is highly expressed in HUVEC exposed to HDP. Moreover, both overexpression and suppression of miR-196a-5p resulted to a significant decrease in the expression of *PDGFRA*, *VEGF*, and *bFGF* genes, highlighting the role of miR-196a-5p in regulating angiogenesis. Therefore, this miRNA, together with *VEGF*, *bFGF*, and *PDGFRA* suggests involvement in the PI3K-AKT pathway. This provides a strong basis for further investigation into the mechanistic role of miR-196a-5p in endothelial cell angiogenesis.

## Figures and Tables

**Table 1:** Overview of the clinical characteristics of the mother and offspring.

Characteristics	Normal HUVEC (n = 8)	HDP HUVEC (n = 8)
Mother		
Age (median, year)	34 (29–39)	35 (24–38)
Race		
Malay	7 (87.6%)	4 (50.0%)
Chinese	1 (12.4%)	3 (37.5%)
Indian	-	1 (12.5%)
Gravida, Para		
G1P0	2 (25%)	2 (25%)
>G1P0	6 (75%)	6 (75%)
Family history with hypertension	4 (50%)	3 (37.5%)
Age of pregnancy when diagnosed with HDP	-	22 weeks
Cesarean delivery	-	3 (37.5%)
Symptom of preeclampsia	-	High BP Urine albumin
Offspring		
Age pregnancy (Mean ± SEM)	38.29 ± 0.57	34.67 ± 1.42
Weight after delivery, kg (median)	2.89	2.49
Complication during delivery	-	Preterm, Small for gestational age (SGA), low body weight

**Table 2:** Details of the products used for miR-196a-5p mimic and inhibitor transfection in HUVEC.

Product	Modification	Sequence
miRCURY LNA™ miRNA Mimic (hsa-miR-196a-5p)	5′-FAM	5′ UAG GUA GUU UCA UGU UGU UGG G
miRCURY LNA™ miRNA Mimic Control	5′-FAM	UCA CCG GGU GUA AAU CAG CUU G
miRCURY LNA™ miRNA Power Inhibitor (hsa-miR-196a-5p)	5′-FAM	5′ UAG GUA GUU UCA UGU UGU UGG G
miRCURY LNA™ miRNA Power Inhibitor Control	5′-FAM	TAA CAC GTC TAT ACG CCC A

**Table 3:** List of target genes primers.

Gene	Title 2	Primer Sequence (5′-3′)	Product Size (bp)	NCBI Reference
*PDGFRA*	Forward	ACTGTTGGAGCTACAGGGAGA	102	NM_001347828.2
	Reverse	TCCGCAATGAATGTCCCACA		
*VEGF*	Forward	AAGGAAGAGGAGACTCTGCGCAGAGC	208	ON777792.1
	Reverse	TAAATGTATGTATGTGGGTGGGTGTGTCTACAGG		
*bFGF*	Forward	CCCCAGAAAACCCGAGCG	109	NM_002006.6
	Reverse	CGCGGCGTCACATCTTCTAC		
*GAPDH*	Forward	TTCTTTTGCGTCGCCAGCC	236	NM_002046.7
	Reverse	TCCCGTTCTCAGCCTTGACG		

## Data Availability

The original contributions presented in this study are included in the article/Supplementary Material. Further inquiries can be directed to the corresponding author.

## Supplementary Materials

The following supporting information can be downloaded at https://www.mdpi.com/article/10.3390/ijms27042047/s1.
