# Supplementary material for: Regulatory Role of miR-196a-5p in Angiogenesis-Related Markers in Endothelial Cells Exposed to Hypertensive Pregnancies
Source: Int J Mol Sci. 2026 Feb 22;27(4):2047. doi: 10.3390/ijms27042047 (PMC12940203; doi:10.3390/ijms27042047)
Supplement: Supplementary file 1 [file ijms-27-02047-s001.zip › Figure S1.pdf]

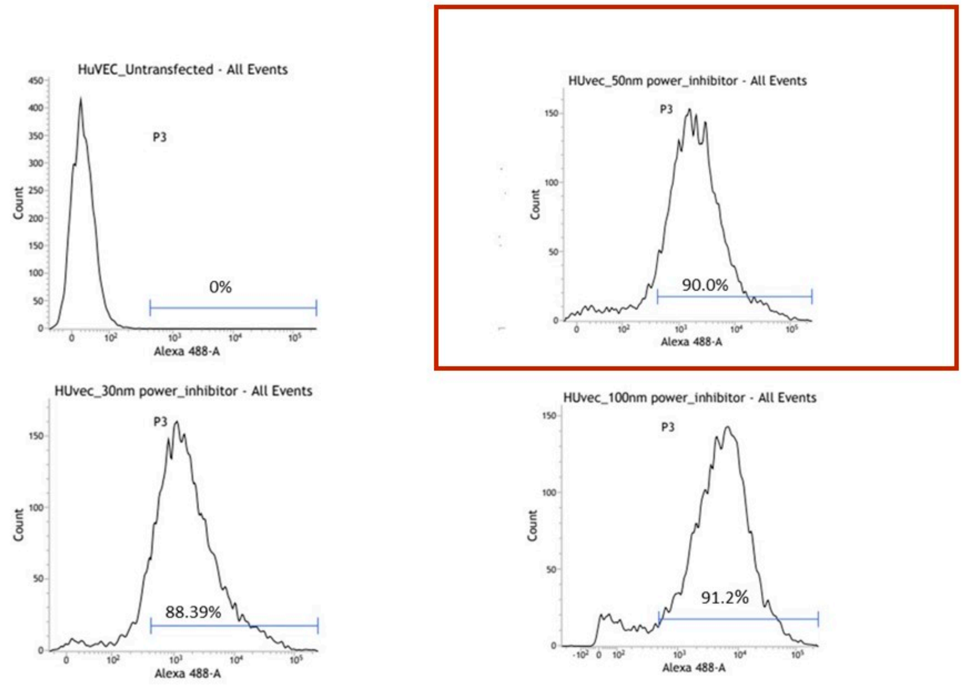

Figure S1. Histogram graph showing transfection efficiency to determine the inhibitor dose using flow cytometer (BD FacsCanto II). The dose of miRNA inhibitor selected was 50nM after showing 90% transfection efficiency on hypertensive HUVEC cells.
